# Adoption of a Short-Term (4-Week) Vegan Diet as Part of ‘Veganuary’ Significantly Reduces Saturated Fatty Acid (SFA), Cholesterol, B12, and Iodine Intake in Omnivorous Individuals—An Observational Study

**DOI:** 10.3390/nu15234967

**Published:** 2023-11-30

**Authors:** Elizabeth Eveleigh, Lisa Coneyworth, Jim Craigon, Simon Welham

**Affiliations:** 1 Division of Food, Nutrition and Dietetics, School of Biosciences, The University of Nottingham, Sutton Bonington LE12 5RD, UK; b.eveleigh@ucl.ac.uk (E.E.); lisa.coneyworth@nottingham.ac.uk (L.C.); 2 School of Biosciences, The University of Nottingham, Sutton Bonington LE12 5RD, UK; jim.craigon@nottingham.ac.uk

**Keywords:** vegan, micronutrients, iodine, plant-based, Veganuary

## Abstract

Global veganism campaigns like ‘Veganuary’ have gained popularity. We conducted an observational study to assess the impact of a 4-week vegan diet during ‘Veganuary’ on nutrient intake, status, knowledge, and motivations for veganism. Data were collected before and after ‘Veganuary’, using Food Frequency Questionnaires (FFQs) to estimate dietary intake. Micronutrient knowledge and motivation were assessed through questionnaires. A total of 154 UK adults aged 18–60 years (2019: *n*81; 2020: *n*73) participated. Groups were vegetarians and omnivores committed to a 4-week vegan diet during ‘Veganuary’. Control groups were vegans and omnivores who did not transition. Short-term vegan diets significantly decreased intake of iodine, B12, cholesterol, and saturated fatty acids (SFAs) in omnivores. Micronutrient knowledge was low, and motivation for veganism varied. Short-term vegan diets reduce macro- and micronutrient intake in omnivores. Veganuary participants could benefit from nutritional guidance or supplementation. Attention is required for UK micronutrient intake and knowledge. Motivations for vegan pledges may influence diets, warranting further research.

## 1. Introduction

Vegan diets are typically defined by the absence of animal products and are predominantly plant-based [1]. Their popularity has increased in recent years, and in 2022, 2,016,600 people in the UK reported their dietary preference to be vegan [2]. Motivations to implement vegan diets have been widely studied [3,4,5,6,7,8,9]. Health improvement is the top reason for reducing animal intake for those who do not consider themselves full-time vegans, whereas permanent vegans tend to have ethical motivations [4,5,9].

Short-term vegan diets may be more appealing and potentially more realistic than permanent veganism due to the flexible nature of dietary intake [9]. Worldwide, there is increasing interest in campaigns that promote temporary or short-term removal of animal products from the diet such as ‘Meat-free Mondays’ or ‘Veganuary’ [9,10,11]. In 2021, more than 500,000 people worldwide signed up for the ‘Veganuary’ campaign and pledged to follow a vegan diet for one month [11].

Well-planned vegan diets can be useful for improving health and minimizing the progression of chronic disease states and are now accepted by the British Dietetic Association (BDA) and other societies worldwide as providing a generally adequate nutrient supply [12,13]. However, any diet that excludes certain food groups may pose a greater risk for micronutrient deficiencies [14,15,16,17,18,19]. Those excluding animal-derived foods (meat, fish, milk, and eggs), may compromise their intake of several micronutrients including vitamin B12, calcium, zinc, iron, and vitamin D [20,21,22,23,24], in addition to those vital for thyroid hormone synthesis such as iodine, selenium, and vitamin A [25,26,27]. The bioavailability of micronutrients from plant foods is often lower than for animal products due to the presence of anti-nutritional factors that reduce or inhibit absorption [28]. In addition, vegan food products imitating animal products may not yet offer comparable micronutrient profiles, are not regularly fortified, and may not provide the food matrix permitting nutrients to be easily absorbed [29,30,31,32,33].

Research into the effects of short-term vegan diets on micronutrient intake is limited [34,35,36]. One study found that short-term vegan diets significantly reduced intake of iodine and vitamin B12 despite participants receiving support from trained nutritional professionals [35]. This raises the question as to whether individuals can adequately plan a short-term vegan diet without expert guidance, particularly when knowledge of micronutrients is low [37,38]. Similarly, data modeling studies have demonstrated that individuals exchanging animal foods for alternative plant substitutes are likely to have a lower micronutrient intake [33].

There is yet to be a study investigating the influence of short-term vegan diets on micronutrient nutrition in individuals who choose to change their diet voluntarily. In this study, we evaluate the change in food consumption between dietary groups before and after pledging to a vegan diet as part of ‘Veganuary’. We address the motivations, attitudes, and perceptions that may influence the decision to adhere to vegan diets. We hypothesized that those following short-term vegan diets would have lowered micronutrient intake, attributed to changing dietary composition and exclusion of animal products when compared to dietary groups not changing their diet. We additionally hypothesized that the decline in micronutrient intake would be less severe for individuals who were already vegetarian compared with omnivores on the basis that vegetarians may have a better understanding of nutritional requirements, as they already have some experience in dietary transition. Similarly, we hypothesized that baseline micronutrient knowledge would be lower in omnivorous participants compared to vegetarian and vegan groups.

## 2. Materials and Methods

UK-wide recruitment was conducted in November–December 2019 and 2020. Flyers and email advertisements were used for those living in Nottinghamshire in 2019. In both years, social and traditional media were used to publicize the study nationally. In 2020, due to the Severe Acute Respiratory Syndrome Coronavirus 2 (SARS-CoV-2/COVID-19) pandemic, all recruitment was conducted online. Criteria for inclusion were adults aged 18–60 years (y) living in the UK. Those recruited either were changing their current diet to follow a vegan diet for 4 weeks as part of the ‘Veganuary’ pledge or were continuing their current diet (e.g., vegetarian, omnivore, etc.). Individuals who were already following a vegan diet were recruited to act as a control.

**Figure 1:**
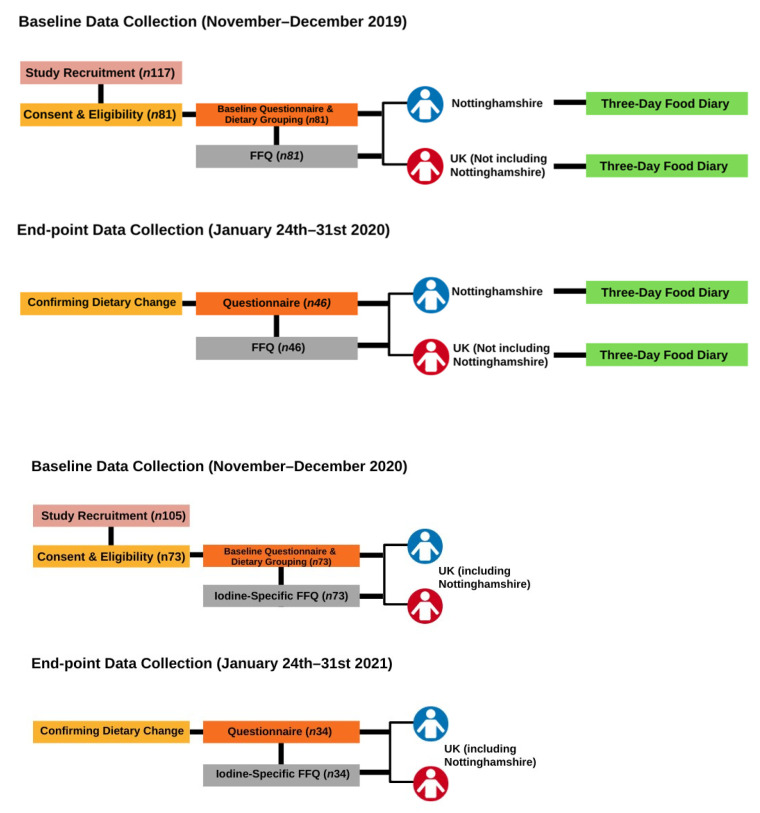
Schematic diagram of the study protocol in years 2019 and 2020. The end of study questionnaire includes questions about attitudes and the food choice questionnaire (FCQ). FFQ, Food Frequency Questionnaire.

The exclusion criteria included those who did not provide consent, pregnant or lactating women, presence of a thyroid disorder, those taking thyroid medication, or having a history of thyroid problems. All participants gave informed consent online before the study. Consent and applicability were assessed by researchers according to study criteria. All participants gave their informed consent for inclusion before they participated in the study. The study was conducted in accordance with the Declaration of Helsinki. Ethical approval was obtained from the Faculty of Medicine and Health Sciences research ethics committee (FMHS REC ref no 391-1909). Following acceptance, participants were provided with a unique participant identification number for further correspondence to prevent bias. A flow diagram of the study methodology is depicted in Figure 1. The study protocol was adapted from that used for the 2019 cohort to allow online participation during the COVID-19 pandemic in 2020. Changes to data collection will be further outlined in the sections below. We have adopted the STROBE guidelines for reporting observational cohort studies in reporting this study [39].

### 2.1. Baseline Questionnaire

An online baseline questionnaire, designed using Online Surveys [40], was completed by eligible participants. Demographic questions addressed age, sex, weight, height, current education, and nationality. In 2020, individuals were also asked about their job circumstances due to the COVID-19 pandemic, e.g., furloughed, working from home, etc. Both surveys (2019 and 2020) asked about health habits and behaviors such as supplement intake, smoking status, and alcohol consumption. Physical activity levels were assessed using questions taken from the self-administered Short International Physical Activity Questionnaire (IPAQ-SF) [41]. Knowledge of the role of micronutrients in the diet was also examined in 2019. In 2020, micronutrient questions were adapted to specifically address iodine knowledge. The iodine knowledge questionnaire was revised from a previously developed and validated version, which provided a total knowledge score ranging from 0 to 8 [42]. Two additional questions were added addressing whether individuals planned their diet to include sources of iodine and asked if any information on iodine was currently being received. The questions asked to assess micronutrient and iodine knowledge are shown in Tables S1 and S2.

### 2.2. Dietary Grouping

Participants were self-allocated into dietary groups according to responses to two questions in the online questionnaire. The first addressed the exclusion of specific food items from the diet, e.g., cow’s milk, poultry, etc. Next, participants selected the dietary group that best described their current diet from a list of possible classifications outlined by Phillips (2005). As definitions of vegan and vegetarian dietary preferences are often inconsistent [1], we additionally asked participants to state their frequency of consumption of animal products in an FFQ. This methodology could not account for recent changes in dietary preference (<6 months) and participants were therefore asked how long they had been following their current dietary preference for clarification. An ‘other’ category was included to outline requirements if not listed. Participants were also asked to define any food intolerances or allergies and were asked if these were professionally diagnosed.

Individuals were asked if they were pledging to follow a short-term vegan diet for January as part of ‘Veganuary’ or were intending to follow their regular dietary preference. The groups included vegans who were remaining vegan (VV), vegetarian-vegans (VegV) who were vegetarians pledging to follow a short-term vegan diet, omnivores who were remaining omnivorous (OO), and omnivore-vegans (OV) who were omnivores pledging to follow a short-term vegan diet. The control groups (VV and OO) were included to permit comparison and to reduce bias. We were unable to establish a solely vegetarian group due to a lack of participation.

### 2.3. Motivation Survey

The baseline attitude questionnaire comprised 23 items to assess motivation for following a vegan diet. For each category (e.g., “health”), respondents were asked to decide how much influence it evoked on a scale of 1 to 5 (1: “no influence at all” to 5: “very strong influence”). Open-ended questions were used to identify additional factors that may have influenced their decision to follow a vegan diet but were not included in the traits listed. This survey was in accordance with published work used to assess motivations for following a vegan diet [8]. Higher mean scores relate to greater motivation for each trait.

### 2.4. Food Choice Survey

A food choice survey assessed the importance of factors influencing food selection and the perceived difference between diets followed at baseline and at the end of January. Questions were designed based on the ‘The Food Choice Questionnaire (FCQ)’ [43,44,45], with items categorized into groups including health, mood, convenience, sensory appeal, natural content, price, weight control, familiarity, and ethical concern. In 2019, the survey contained 28 items. In 2020, the survey contained 24 items. For both years, a five-point Likert scale was used to score responses ranging from 1 (“Strongly Disagree”) to 5 (“Strongly Agree”). Higher mean scores indicate greater importance of a particular factor in influencing food choice.

### 2.5. Dietary Adherence Log

To track the contribution of animal product consumption (accidental or deliberate) to micronutrient intake in VegV and OV groups during the study, we developed an online dietary adherence log called “whoops”. Participants were asked to record the date of consumption, the amount of food consumed, and a brief description of the circumstance. Drop boxes were provided to allow individuals to select the item that was consumed. If participants did not want to fill in the log, they were asked to contact researchers in the event of animal consumption.

### 2.6. Food Frequency Questionnaire (FFQ)

We used an online FFQ survey which included a 139-item semi-quantitative FFQ adapted from the European Prospective Investigation into Cancer and Nutrition Norfolk FFQ (EPIC-Norfolk FFQ) [45,46]. Modifications were made to improve applicability to the population of interest, including the addition of food groups and imitation products regularly consumed by individuals avoiding animal products, accounting for foods such as dairy-free alternative milk, cheeses, creams, dressings, spreads, and alternative meats. The FFQ additionally addressed the brand and type of dairy-free milk consumed, including fortification status and storage of the product, to improve the estimation of the relative contribution to micronutrient intake. The FFQ administered at baseline was designed to assess dietary intake over the past 6 months. The FFQ provided at the end of the study in January was modified to record a month of dietary intake. The duration of the recording was selected to benefit those who only recently changed their dietary preference, to improve recall, and to account for new food product launches in UK supermarkets. To minimize portion size as a source of error, images, and portion size guides of average portions of foods regularly consumed in the UK diet were included. We did not ask about the consumption of sauces or preserves and did not differentiate between homemade and retail cakes and pies to minimize participant burden.

The FETA software (Version 6) was used to calculate nutrient values. The contribution of select vegan and vegetarian food products is not included in the FETA software. The original FETA software only contains 9 milk codes, of which only one is dairy-free (soya) and this generic soya code does not contain fortification details. We modified the FETA look-up files to account for 16 new food groups added to our FFQ along with 7 new non-dairy alternative milks. Nutrition values and portion sizes for these additions were generated from food items listed on Nutritics software (Research Edition Version 5) (www.nutritics.com/en/ (accessed on 9 November 2019)) or were set to match similar foods already present in FETA look-up files.

The program allows only one type of milk; therefore, only the dominant milk choice was used if participants consumed semi-skimmed cow’s milk and soya milk. The relative consumption of each milk was calculated, and the milk consumed in the greatest quantity was coded. For participants who did specify the brand and type of dairy-free alternative milk consumed, the generic food code for ‘soya milk’ was selected. Milk frequency was quantified according to measures listed within FETA, e.g., quarter of a pint, half a pint, etc., calculated from FFQ outputs. Our FFQ did not include specific questions about cereal consumption; therefore, a non-specific cereal code ‘a1362′ was selected. Additionally, we did not record the type of frying or baking fat used. The default fat codes ‘17046′ and ‘17018′ were selected. Nor did we ask about visible fat; the code ‘-9′ was used. Foods that were not considered in the FFQ, as mentioned above, were automatically coded as ‘Never as or less than once a month’.

Participants were instructed to document their daily supplement intake in the FFQ, specifying the brand and type of supplement. However, they were not queried about consumption frequency, so regular intake was presumed. To determine the micronutrient content of these supplements, brand information was identified online from retailers’ websites. The supplement content was then integrated with nutrient estimates obtained from the FFQ.

### 2.7. Statistical Analysis

Analyses were conducted using Genstat (20th edition) and SPSS with a significance level set at *p* ≤ 0.05. Data were checked for normality; where data were not normally distributed, non-parametric tests were conducted and values were presented as median (75th percentiles). Non-skewed data were analyzed using parametric tests and expressed as mean (SD). A general ANOVA balanced design with factors “Time” and “Treatment” was used to assess the effect of short-term vegan diets on all measures of micronutrient intake and status. “Time” describes the point at which data were collected, either before or after January, and “treatment” describes the dietary group (VV, VegV, OO, and OV). In our analysis, “Treatment”*“Time” were used as factors with participant number (ID) as a blocking factor. The Fisher–Freeman–Halton Exact Test was used to assess significance between dietary groups for counts (correct scores) of micronutrient and iodine knowledge. The 2019 and 2020 cohorts were analyzed and considered separately; this is because the study design and outcomes measures were not directly comparable. Additionally, variations in food intake may have occurred between the cohorts due to food availability and personal circumstances experienced during the COVID-19 pandemic. Participants with incomplete data (missing data) at either baseline or at the end of the study were excluded from analysis where two sets of data were required (e.g., food intake). Individuals who were lost before the end of the study were still able to provide data for outcomes that were only recorded at baseline (e.g., motivation). The sample size was calculated from our previous data (UIC values), which showed that for those who were already established vegans, the degree of difference in iodine status compared to omnivores was approximately 16 μg L^−1^ (78 μg L^−1^ vs. 62 μg L^−1^) with a standard deviation of approximately 30 μg L^−1^ [38]. With these values, we would expect to require a sample size of ~56 participants in total to see a statistically significant difference (*p* ≤ 0.05).

## 3. Results

### 3.1. Participant Demographics

**Figure 2:**
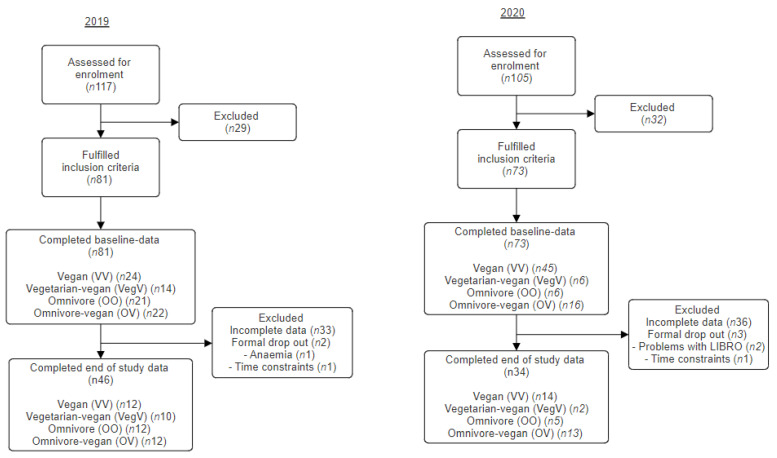
Study flowchart.

Following screening and confirmation of eligibility, 154 UK adults participated in the study at baseline (2019; *n*81, 2020; *n*73). In both years, the attrition rate was high, with 40% failing to provide data at the end of January (end of study) in 2019 and 49% in 2020. The formal dropout rate was 2% in 2019; reasons for not continuing were newly diagnosed anemia and time constraints. In 2020, this rate increased to 4%. The main reasons for dropping out of the study in 2020 were difficulties recording food intake along with challenges with participation caused by the COVID-19 pandemic lockdown restrictions (Figure 2). Participant demographics are presented in Table 1.

### 3.2. Motivations to Follow a Vegan Diet

For those already following a vegan or vegetarian diet, their dietary choice in both years was strongly driven by issues with factory farming and animal welfare (*p* < 0.01; Tables S3 and S4). This was also true for omnivores choosing to try a vegan diet but to a lesser extent. Sustainability and climate protection were more important for vegans and vegetarians compared with omnivores in 2019 (*p* < 0.001), but these were more even across dietary groups in the 2020 cohort (*p* = 0.500 and *p* = 0.301, respectively). For both omnivore groups, health was the top motivational factor in 2019, but this changed to climate and sustainability in 2020.

### 3.3. Dietary Intake by Food Frequency Questionnaire in 2019

**Figure 3:**
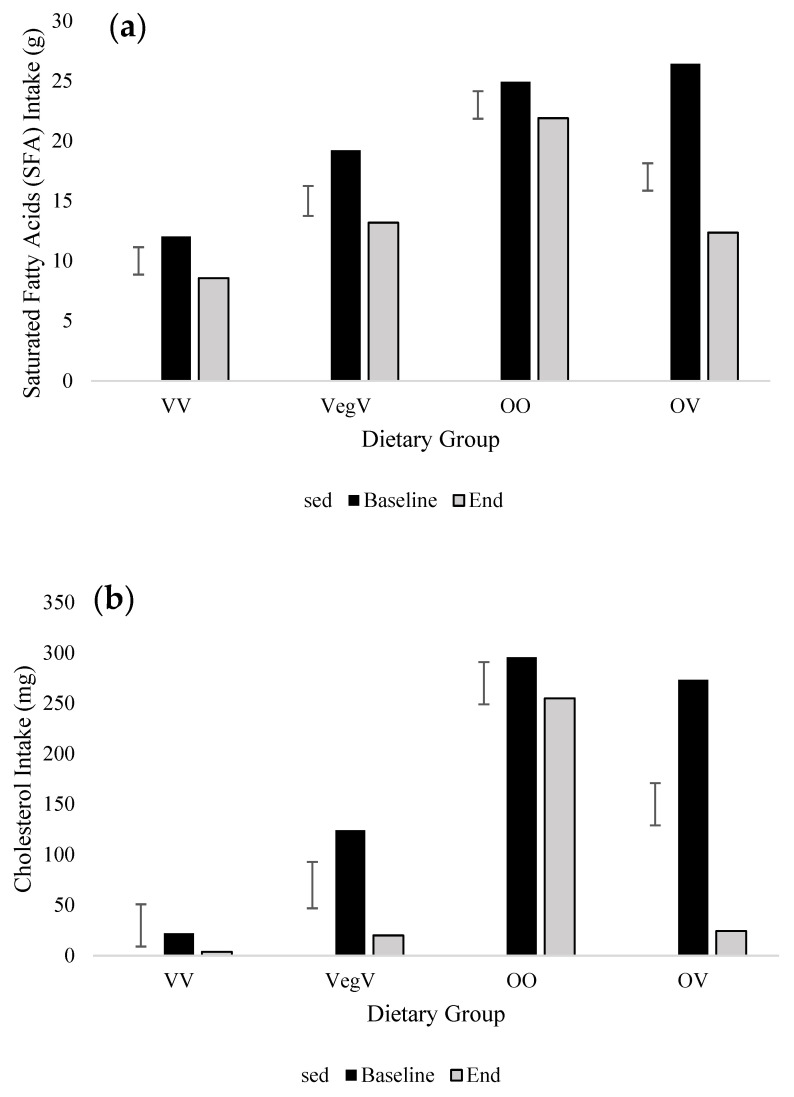
Average daily macronutrient intake non-inclusive of supplements of individuals in the 2019 cohort at baseline and end of study (End) provided Food Frequency Questionnaire (FFQ) estimates. (**a**) Saturated fatty acid intake (SFA), (**b**) cholesterol intake. Mean and standard error of the difference (within-subject) presented (SED). Only significant effects are presented and assessed by ANOVA (*p* ≤ 0.05). Groups are VV, vegan (*n*12); VegV, vegetarian-vegan (*n*10); OO, omnivore-omnivore (*n*12); and OV, omnivore-vegan (*n*12).

**Figure 4:**
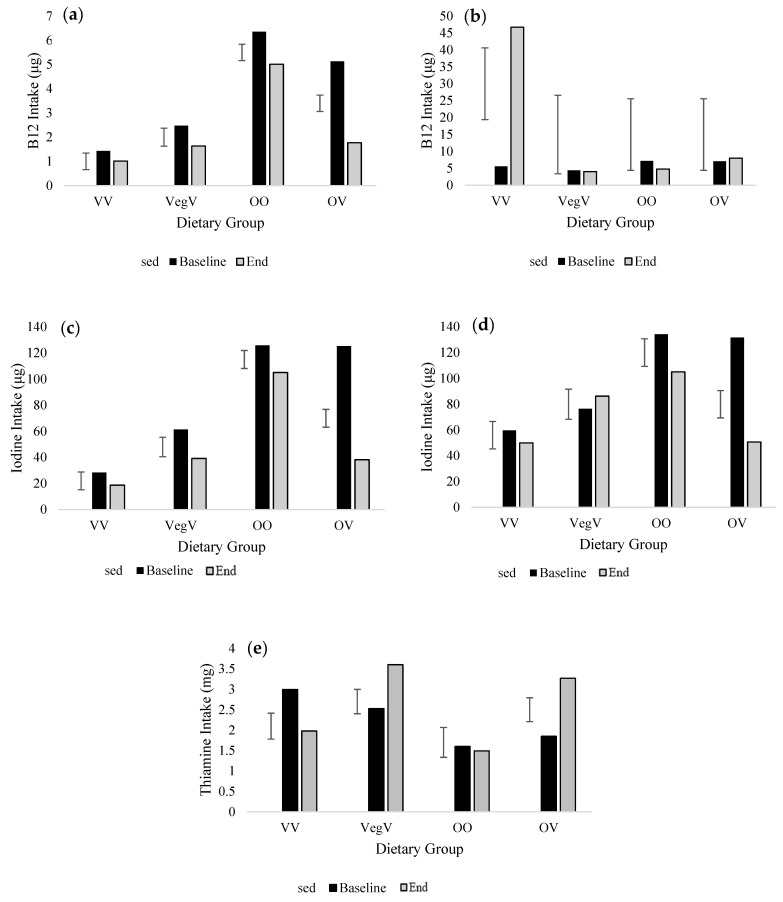
Average daily micronutrient intake non-inclusive and inclusive of supplements of individuals in the 2019 cohort at baseline and end of study (End) provided Food Frequency Questionnaire (FFQ) estimates. (**a**) Vitamin B12 intake non-inclusive of supplements, (**b**) vitamin B12 intake inclusive of supplements, (**c**) iodine intake non-inclusive of supplements, (**d**) iodine intake inclusive of supplements, (**e**) thiamine intake non-inclusive of supplements. No significant effects were found for thiamine intake inclusive of supplements. Mean and standard error of the difference (within-subject) presented (SED). Only significant effects are presented and assessed by ANOVA (*p* ≤ 0.05). Groups are VV, vegan (*n*12); VegV, vegetarian-vegan (*n*10); OO, omnivore-omnivore (*n*12); and OV, omnivore-vegan (*n*12).

FFQ data clearly showed both positive and negative effects of the transition to a vegan diet (Table 2 and Table 3). Saturated fat (SFA) intake was lower in vegans compared with other groups (*p* ≤ 0.001) and was shown to be reduced by 10.6 g and 14.0 g in vegetarians and omnivores, respectively, when transitioning to a vegan diet (*p* = 0.005; Figure 3). Cholesterol intakes exhibited considerable reductions with diet and time point. Vegans consumed roughly 1/10th of that consumed by omnivores, who themselves showed a 10-fold reduction in intake when adopting a vegan diet (*p* = 0.001; Figure 3). Vegetarians transitioning to a vegan diet similarly reduced their cholesterol intake by 104.1 mg. Before the addition of supplements to the analysis (Figure 4), consumption of vitamin B12 was significantly lower in vegans and vegetarians (*p* < 0.001) and was reduced by 3.3 μg in omnivores changing to a vegan diet (*p* < 0.001). After the inclusion of supplements (Figure 4), B12 intake substantially improved in the VV group at the end of the study (5.6 μg to 46.7 μg). Additionally, there were improvements in B12 for the OV group, such that changing to a vegan diet no longer presented a significant reduction in intake over time (*p* = 0.422; Figure 4). Iodine intake (non-inclusive of supplements) decreased in all groups between December and January but was considerably reduced in omnivores transitioning to a vegan diet (*p* < 0.001; Figure 4). There remained a significant reduction in iodine intake in omnivores transitioning to a vegan diet once supplements were added to the analysis (*p* = 0.033; Figure 4). At both time points, iodine intake also improved with the addition of supplements in VV and VegV groups, but average intake remained below the RNI (140 µg day^−1^). Thiamine intake significantly increased in omnivorous individuals transitioning to a vegan diet (OV) from baseline to end, rising from 1.9 mg to 3.3 mg (*p* = 0.020; Figure 4).

**Figure 5:**
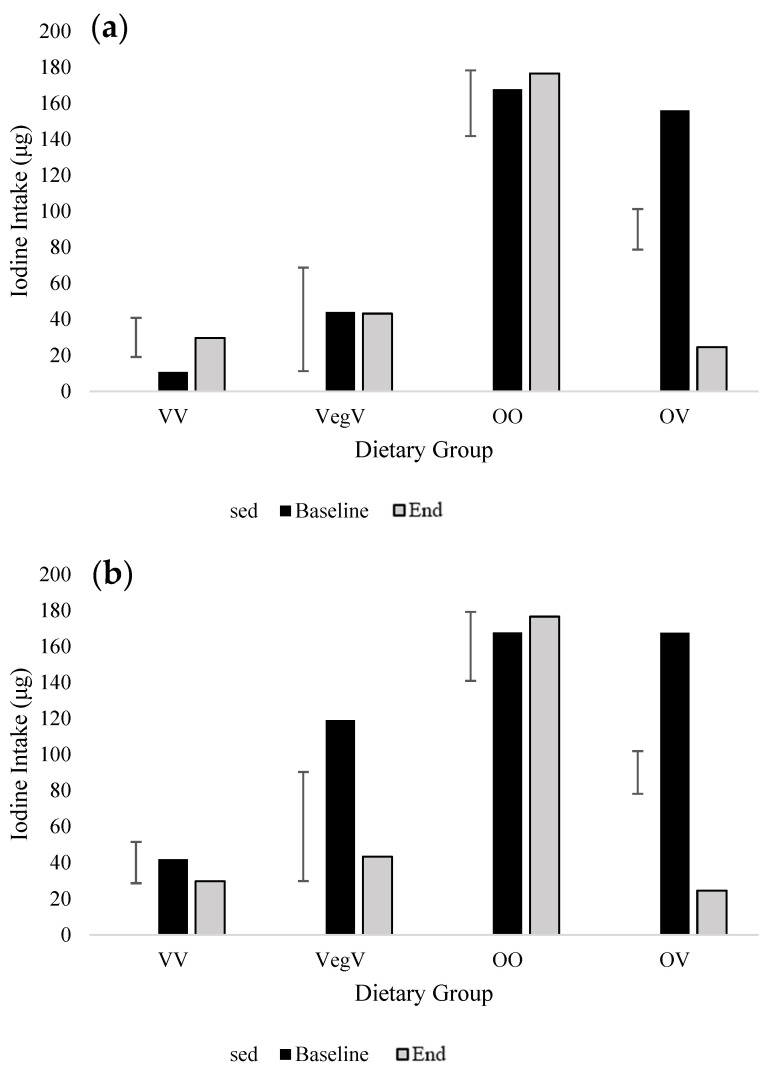
Average daily iodine intake non-inclusive and inclusive of supplements of individuals in the 2020 cohort at baseline and end of study (End) provided iodine-specific Food Frequency Questionnaire (FFQ) estimates. (**a**) Iodine intake non-inclusive of supplements, (**b**) iodine intake inclusive of supplements. Mean and standard error of the difference (within-subject) presented (SED). Significant effects are presented and assessed by ANOVA (*p* ≤ 0.05). Groups are VV, vegan (*n*14); VegV, vegetarian-vegan (*n*2); OO, omnivore-omnivore (*n*5); and OV, omnivore-vegan (*n*13).

For the 2020 cohort, we felt that the emotional burden of the continued COVID-19 lockdown, particularly with the swift Christmas shutdown, might reduce the level of participation and increase attrition, so the FFQ was truncated to measure iodine intake only. Like in 2019, iodine intake (non-inclusive of supplements) showed a significant decrease (131.4 µg) over time for the OV group (*p* < 0.001; Table 4; Figure 5).

The greatest reduction in iodine was from loss of cow’s milk from the diet (38.4 μg less than pre-vegan January intake), followed by dairy yogurt (21.7 μg less) and white fish (9.7 μg less). There was additionally a significant increase in iodine provided by dairy cheese in the OO group from baseline to end (*p* < 0.001). Dietary supplements did not increase the iodine intake of individuals transitioning to a vegan diet in January (*p* = 0.001; Figure 5).

### 3.4. Supplement Intake

For participants in 2019, consumption of supplements at baseline was high, with >80% in the VV group, ~50% in the VegV and OO groups, and ~60% in the OV group (Table S5). Vitamin B12 was most frequently supplemented in VV (50% of supplements) and VegV groups (60%). Individuals in the VV group also often consumed multivitamin supplement complexes (50%). Vitamin D was the most consumed supplement by OO (50%) and OV groups at baseline (27%).

By the end of the study, fewer VV individuals recorded consuming supplements (66%, down from 83%), while the proportion of VegV taking supplements increased by 80%, OV by 16%, and OO declined by 77%. Vitamins B12 and D remained the most consumed supplement for participants in the VV and OO groups (~75%). For the VegV group, the number of people consuming multivitamin supplement complexes increased from 20% to 90% at the end of the study. The number of people taking Vitamin B12 supplements also increased in the group from 10% to 60%.

Participants from the 2020 cohort showed broadly similar levels of supplement usage to those seen in 2019 (Table S6). Although none of the OO group in 2020 recorded any supplement intake, numbers in this group were low (*n*5).

Reasons for taking supplements were predominantly due to following a diet that does not contain animal products amongst vegans and vegetarians, whilst the principal reasons for supplementation in the OV group were to improve health and due to a lack of sunlight exposure in the UK winter.

### 3.5. Food Choice Survey

Factors of food choice varied by dietary group in 2019 (Figure S1). The strongest determinant of food choice for omnivores and vegetarians was sensory appeal, with price also being similarly important for vegetarians specifically. For vegans, familiarity was the strongest driver of choice. Weight control was the lowest-scored factor of food choice for all omnivores and vegans, whilst for vegetarians, convenience was the least important. Vegans placed health, mood, and familiarity significantly higher than did omnivores transitioning to vegan, for whom price, weight control, and ethical reasons were significantly lower than for vegetarians (*p* = 0.001). We were unable to include an assessment of food choice for those in the VegV group due to low numbers (*n*2) in 2020. The OO group ranked mood as the strongest determinant of food choice, whereas naturalness was the strongest determinant for the OV group and price for the VV group. The OO group had significantly higher scores than the OV group for determinants of mood, price, and “ethical”, at a significance level of *p* = 0.05.

### 3.6. Micronutrient Knowledge

Overall, micronutrient knowledge at baseline was low (Table S7). Fewer than 30% of respondents from each dietary group were aware of micronutrients in general. Awareness was lowest for micronutrients niacin and vitamin B6. Iron (VV, 54%; VegV, 53%; OO, 35%; OV, 43%) and vitamin D (VV, 46%; VegV, 33%; OO, 50%; OV; 48%) were most frequently recognized by participants. Individuals in the VV group had significantly greater awareness of vitamin B12 than those in the OV group.

Over half of the participants were able to describe a symptom of micronutrient deficiency (VV, 58%; VegV, 67%; OO, 55%; OV, 65%). Symptoms of iron deficiency (anemia, fatigue, etc.) and vitamin D deficiency (bone health, rickets, etc.) were most often described. Participants had low awareness of symptoms of deficiencies in folate, calcium, magnesium, and zinc. Awareness of symptoms of vitamin B12 deficiency was significantly higher in the VV group (25%) than in the OV group (0%).

### 3.7. Iodine Knowledge

Iodine knowledge was poor in the 2020 cohort, with an average knowledge score of 2 out of a possible total of 8 (Table S8). At baseline, the omnivore group had significantly greater iodine knowledge compared to the VV group, with a score of 3.0 vs. 1.4 (*p* = 0.004). There were significant differences in the knowledge of iodine deficiency being a problem in the UK, of dietary planning to achieve adequate iodine intake, and receipt of iodine information between dietary groups.

## 4. Discussion

In recent years, there has been growing interest in part-time or short-term veganism. The ‘Veganuary’ challenge is one example of this shift to temporary dietary change and may be adopted for health reasons, as a challenge, or for a more manageable way of reducing animal products in the diet [11]. A well-planned vegan diet can meet dietary recommendations [12]; however, studies have found that certain micronutrients may be of concern in those following a vegan diet [15,16,18,47,48,49]. To our knowledge, this study is the first to investigate the change in micronutrient intake and status in those choosing to follow a short-term (4 weeks) vegan diet as part of ‘Veganuary’.

Our study shows that the adoption of a short-term (4-week) vegan diet has a significant effect on the nutrient intake of individuals following an omnivorous diet, particularly in those who were not consuming dietary supplements, but not vegetarians. This is likely to be due to our vegetarian participants recording a very low micronutrient intake at baseline and consuming dietary patterns that were more similar to vegans than omnivores before the study. This finding agrees with research from other studies that have recorded comparable micronutrient intake in vegetarians and vegans [18,38,50,51,52].

SFA and cholesterol intake significantly decreased in omnivores pledging to a short-term vegan diet during Veganuary. Fat reduction observed in omnivores in our study was similar to differences reported in previous studies [53,54,55,56]. Vegans typically have a lower intake of SFA and cholesterol compared to omnivores due to the absence of meat, dairy, fish, and eggs from the diet [16,57]. However, SFA and cholesterol can also be found in plant foods such as alternative products, vegetable oils, coconut, nuts, and chocolate [58]. We did not assess the top sources of SFA and cholesterol in the diet at baseline or at study end; however, the replacement of animal products with plant-based foods has likely contributed to lower intake in the OV group [50,59,60]. Reduced intake of SFA and cholesterol have been shown to decrease cardiometabolic risk and body weight [59]. Short-term vegan diets may therefore be appropriate for people aiming to reduce their SFA and cholesterol intake.

Thiamine intake significantly increased with time for omnivores following a short-term vegan diet. This result does agree with other studies [48]. Thiamine intake is not usually a problem in vegan diets, as plant foods such as fortified cereals, whole grains, beans, and pulses are rich sources [61].

Lower vitamin B12 intake has been frequently recorded in vegan and vegetarian populations [20,21,22,23,24]. Vitamin B12 is synthesised by bacteria colonizing the gastrointestinal (GI) system of animals, and once produced, it is stored in the tissue of the host. For this reason, vitamin B12 can only be obtained from animal foods [62]. We found a significant reduction in B12 intake in non-supplementing omnivores following a short-term vegan diet, matching the results of other studies [63]. Many plant foods are now fortified with vitamin B12, including alternative milk, dairy products, and meats, along with some breakfast cereals and yeast products [23]. Values for vegan diets may potentially be lower because some fortified sources of vitamin B12 may not have been captured by our FFQ, but the majority are present, and therefore we feel the values provide a good estimate. Supplements have proven to be a useful way to improve vitamin B12 intake in those following vegan and vegetarian diets [20]. After the inclusion of supplements, we found that B12 intake substantially improved in the VV group (5.6 μg to 46.7 μg), which may have confounded the other treatment differences observed. Supplements may be consumed daily, episodically, or only during certain circumstances, for example, during illness, hence creating huge variability in estimates [64]. The number of people in the omnivore-vegan group who consumed vitamin B12 supplements in the FFQs increased from 10% at baseline to 60% at the end of the study.

Knowledge and awareness of vitamin B12 were significantly lower in the OV group compared to the VV group. Consequently, individuals in the OV group may be less careful selecting fortified foods or supplements containing vitamin B12. Vitamin B12 can be stored internally (mainly in the liver) and is depleted slowly. Bodily stores of vitamin B12 take 3–4 years to deplete [65]; thus, 4 weeks of reduced intake during Veganuary is unlikely to have a negative influence on vitamin B12 status, unless individuals had low stores before pledging to change their diet. If the dietary transition persists, however, then those who were previously omnivorous may be at greater risk of future B12 deficiency due to a lack of prior knowledge.

Iodine intake also significantly decreased over time in the OV group. The World Health Organization (WHO) criteria for adequate iodine intake indicates that people should consume 150 µg day^−1^ to achieve adequacy [66]. However, in the UK, the RNI for iodine, set in 1991, is lower, at 140 µg day^−1^ [67,68]. As reported in other studies, we found that the iodine provision for UK adults was inadequate, regardless of dietary choice [26,38]. Vegans and vegetarians are frequently recognized as a subgroup at risk of iodine deficiency, since foods typically rich in iodine are mostly of animal origin (cow’s milk, dairy products, eggs, white fish, and seafood) [25,69]. Many vegan alternatives to these foods sold in the UK are not fortified with iodine [30,70]; hence, transitioning to these foods could compromise iodine consumption [29]. In 2020, a cross-sectional study assessed the iodine fortification status of vegan plant-based alternative products (e.g., plant-based milk, dairy, cheese, and seafood) and the impact that substitution with these products could have on dietary iodine [29]. The investigators found that the replacement of three servings of dairy products with non-fortified alternative products reduced iodine delivery by 97.9% [29]. Individuals transitioning to vegan diets in the short term must be aware of the need to select iodine-fortified alternative milk drinks. The FFQs in both years of our study were modified to include information on the fortification status of different alternative milk. In the 2020 cohort (OV iodine intake decreased from 156.0 µg to 24.6 µg), it was apparent that loss of dairy cheese and seafood placed a further burden on iodine intakes on top of that from cessation of milk consumption. Our iodine-specific FFQ had a limited number of items and did not contain foods providing very small quantities of iodine to the diet, e.g., fruit, vegetables, snacks, and/or drinks; therefore, actual dietary iodine may be slightly greater, but this will be very limited. Iodine supplementation was greatest in the OV group at study end, with 23% of participants taking a supplement providing iodine quantities above the RNI for iodine (150 µg day^−1^), which boosted intake in this group, but did not translate to significant improvements in iodine intake [66].

Additionally, our findings show that there are differences in dietary motivations of different groups tracked in our study that could influence food choice and micronutrient intake. Motivation to follow a vegan diet has been widely studied [6,7,9,71], with the majority studying those who have already transitioned and have relatively stable beliefs. In line with other studies [72], we found that environmental motives such as sustainability and climate protection along with ethical and animal-related motives (factory farming and animal welfare) were most important to those following a vegan or vegetarian diet at baseline (VV or VegV groups). Individuals following an omnivorous (OO and OV groups) diet listed health as the biggest motivator. Health motives have been suggested to be adopted by individuals agreeing to immediate self-interest [72]. In terms of our study, it is possible that omnivores may follow vegan diets for personal gain, for example, to provide personal health benefits, e.g., weight loss, improved immunity, etc. [73]. Omnivorous individuals may be less interested in motives that do not directly influence them, e.g., animal- and planetary health-related motives [72]. There are differences in motivations to follow a vegan diet according to age, with younger individuals being significantly more likely to be driven by animal and environmental motives. In our study, individuals in the OV group tended to be older, which may account for the difference in motives between groups [72].

In the 2020 cohort, motivation scores for disease outbreaks for the omnivore-vegan group and scores for COVID-19 infection for vegetarian-vegans were significantly greater than in other dietary groups. During the COVID-19 pandemic, many consumers shifted their consumption from meat products to plant foods [74]. Generally, omnivorous individuals had the lowest motivation scores; this result was to be expected, as motivation to transition to a vegan diet must be low given that these individuals eat animal products.

### Limitations

A major limitation of our study was the low participant numbers and high attrition rate amongst those who began the study. Whilst we observed nutritional differences, it is likely that further differences would have been seen with a larger study population. Future studies will likely require a more thorough advertising approach as well as reducing participant burden to diminish attrition. Additionally, we cannot eliminate the possible seasonal implications of this research in the context of food intake. The study was conducted in November-January, so it is likely that food intake was not representative of the habitual diet over the holiday period and that individuals may be implementing weight loss regimes in January, thus lowering micronutrient intake [75]. A further challenge faced was that the COVID-19 pandemic influenced UK dietary habits significantly in both the initial months of the pandemic and throughout 2020–2021 [74]. Our study did not assess food availability, food security, or other changes in food habits during the COVID-19 pandemic, which may have influenced our results [74]. We were unable to obtain biological samples to enable assessment of biomarkers of iodine status (e.g., urinary iodide and serum thyroglobulin concentrations). This would have been of significant benefit for determining the biological impact of the short-term loss of dietary iodine and will be important to include in any future studies of this type. However, given the novelty of this study, the observation of a significant reduction in certain micronutrients, and the increased uptake of individuals pledging to the Veganuary campaign worldwide, we see scope for this investigation to be completed on a larger number of participants in the future to improve generalizability.

## 5. Conclusions

Our study shows that short-term vegan diets have a significant effect on the intake of vitamin B12, iodine, SFA, cholesterol, and thiamine for individuals who typically follow omnivorous diets. Following a vegan diet in the short term may benefit omnivores wishing to reduce their SFA or cholesterol intake. However, omnivores without experience of consuming a vegan diet may not be able to plan their diet sufficiently well to meet micronutrient requirements. Participants who lack micronutrient knowledge and/or experience in making vegan meals may select unfortified convenience food items and further limit micronutrient intake. Omnivores pledging to vegan campaigns may wish to seek nutritional advice before switching diets to ensure their nutrient intake is adequate. As this is unlikely for many, in the absence of dietary consultation, there is a need for clear guidance that is visible to all those considering such a dietary change. If vegan diets are continued after a short-term trial such as Veganuary, individuals must ensure they are supplementing appropriately or selecting fortified alternative foods to prevent clinical deficiency symptoms. There is a broader need to improve micronutrient knowledge and intake throughout the UK population regardless of dietary preference, and this, alongside a more large-scale assessment of the impact of short-term dietary transition, is warranted.

## Figures and Tables

**Table 1:** Participant characteristics at baseline data collection for 2019 and 2020 cohorts. Values are % prevalence and mean (SD).

	2019				2020			
	Vegan (VV) (*n*24)	Vegetarian- Vegan (VegV) (*n*14)	Omnivore (OO) (*n*21)	Omnivore-Vegan (OV) (*n*22)	Vegan (VV) (*n*45)	Vegetarian- Vegan (VegV) (*n*6)	Omnivore (OO) (*n*6)	Omnivore-Vegan (OV) (*n*16)
Sex *(*%)								
Female	75.0	85.7	76.2	81.8	73.3	100.0	83.3	75.0
Age (Years), Mean (SD)	31.2 (10.4)	35.2 (11.6)	30.10 (9.5)	39.1 (13.8)	26.7 (8.4)	23.3 (3.2)	24.8 (2.0)	34.7 (15.3)
Highest education level (%)								
Secondary (GCSE equivalent)	0.0	7.1	0.0	9.1	2.2	0.0	0.0	0.0
College (A-level equivalent)	16.7	21.4	9.5	0.0	31.1	33.3	16.7	6.2
Undergraduate degree	62.5	35.7	42.9	59.1	46.7	50.0	50.0	31.2
Postgraduate degree (MSc, PhD)	8.3	35.7	42.8	27.2	20.0	16.7	33.3	37.5
Other	12.5	0.0	4.8	4.5	0.0	0.0	0.0	6.2
Employment status (%)								
Employed	50.0	92.9	33.3	59.1	53.4	33.3	33.3	62.4
Furloughed	-	-	-	-	4.4	0.0	0.0	0.0
Student	25.0	0.0	38.1	18.2	28.9	16.7	66.7	25.0
Employed student	16.7	7.1	28.6	22.7	13.3	33.3	0.0	12.5
Unemployed	8.3	0.0	0.0	0.0	0.0	0.0	0.0	0.0
Nationality (%)								
United Kingdom	95.8	92.9	85.7	81.9	-	-	-	-
Europe	0.0	0.0	4.8	9.0	-	-	-	-
East and Southeast Asia	4.2	0.0	4.8	9.0	-	-	-	-
United States	0.0	7.1	4.8	0.0	-	-	-	-
Mexico	0.0	0.0	0.0	0.0	-	-	-	-
Oceania	0.0	0.0	0.0	0.0	-	-	-	-
Ethnicity (%)								
Caucasian—British/Irish	-	-	-	-	86.7	100.0	100.0	87.5
Caucasian—Other	-	-	-	-	11.1	0.0	0.0	6.2
Other	-	-	-	-	2.2	0.0	0.0	6.2
Weight status (BMI) (SD)	22.6 (3.4)	24.1 (7.5)	23.6 (4.3)	26.2 (6.9)	23.8 (4.8)	23.4 (2.0)	21.3 (2.5)	23.6 (3.3)
Minutes per Week of Moderate Physical Activity								
<30	4.2	14.3	15.0	18.2	2.2	0.0	0.0	6.2
30–90	20.8	7.1	5.0	27.3	31.1	33.3	16.7	37.5
90–150	12.5	14.3	30.0	27.3	20.0	0.0	16.7	31.2
150–300	25.0	35.7	30.0	13.6	31.1	16.7	50.0	18.8
>300	37.5	28.6	20.0	13.6	15.6	50.0	16.7	6.2
Smoking status (%)								
Non-smoker	70.8	64.3	95.2	54.5	80.0	100.0	83.3	68.8
Smoker	8.3	28.6	0.0	22.7	6.7	0.0	0.0	6.2
Vaper	-	-	-	-	4.4	0.0	0.0	6.2
Ex-smoker	20.8	7.1	0.0	22.7	8.9	0.0	16.7	18.8
Did not disclose	0.0	0.0	4.8	0.0	0.0	0.0	0.0	0.0
Weekly alcohol consumption (%)								
None	12.5	0.0	14.3	22.7	33.3	33.3	50.0	18.8
<5 Units	41.7	42.9	38.1	31.8	37.8	66.7	33.3	18.8
5–10 units	29.2	28.6	23.8	13.6	13.3	0.0	16.7	31.2
10–15 units	12.5	21.4	19.0	22.7	11.1	0.0	0.0	18.8
15+	0.0	7.1	0.0	9.1	4.4	0.0	0.0	12.5
Prefer not to disclose	4.2	0.0	4.8	0.0	0.0	0.0	0.0	0.0
Frequent salt usage (%)								
No	50.0	38.5	33.3	47.6	26.7	83.3	66.7	18.8
Yes, table salt	33.3	46.2	33.3	42.9	44.4	16.7	33.3	43.8
Yes, iodized salt	0.0	0.0	14.3	4.8	6.7	0.0	0.0	6.2
Yes, other salt	16.7	15.4	19.0	4.8	22.2	0.0	0.0	31.2
Length of diet (%)								
0–3 Months	16.7	14.3	9.5	9.1	2.2	0.0	0.0	0.0
3–6 Months	16.7	0.0	0.0	4.5	0.0	0.0	0.0	6.2
6–12 Months	12.5	7.1	4.8	22.7	22.2	16.7	0.0	12.5
12+ Months	41.7	57.1	9.5	9.1	37.8	50.0	0.0	31.2
5+ Years	12.5	21.4	76.2	54.5	37.8	33.3	0.0	12.5
Prefer not to disclose	-	-	-	-	0.0	0.0	100.0	37.5
Dietary intolerance (%)								
None	87.5	85.7	90.5	86.4	68.9	50.0	83.3	100.0
Yes, not professionally diagnosed	12.5	7.1	0.0	13.6	20.0	33.3	0.0	0.0
Yes, professionally diagnosed	0.0	7.1	9.5	0.0	11.1	16.7	16.7	0.0

-: Not applicable.

**Table 2:** Average daily macronutrient intake non-inclusive of supplements of individuals in the 2019 cohort at baseline and end of study (End) provided Food Frequency Questionnaire (FFQ) estimates.

Baseline (Mean)					End (Mean)				SED^(a)^	df^(a)^	SED^(b)^	df^(b)^	*p*-Value (Baseline-to-End)		
	Vegan (VV) (*n*12)	Vegetarian-Vegan (VegV) (*n*10)	Omnivore (OO) *(n*12)	Omnivore-Vegan (OV) (*n*12)	Vegan (VV) (*n*12)	Vegetarian-Vegan (VegV) (*n*10)	Omnivore (OO) *(n*12)	Omnivore-Vegan (OV) (*n*12)	Time × Diet		Time × Diet		Time	Diet	Time × Diet
Energy (kcal)	1275.9	1645.4	1656.3	1720.3	1073.2	1516.4	1514.7	1422.7	149.5	42.0	220.2	65.0	0.010 *	0.083	0.840
Protein (g)	50.4	56.1	76.3	72.5	37.4	56.8	69.4	47.0	7.3	42.0	9.8	70.0	0.002 *	0.008 *	0.087
COH (g)	158.2	193.7	183.2	203.6	141.5	171.5	162.9	204.3	17.9	42.0	25.6	67.0	0.109	0.109	0.778
Fat (g)	50.1	66.7	70.5	69.5	40.7	65.3	63.6	48.3	7.2	42.0	10.5	65.0	0.006 *	0.071	0.253
SFA (g)	12.0	19.2	24.9	26.4	8.6	13.2	21.9	12.4	2.4	42.0	3.3	70.0	<0.001 *	<0.001 *	0.005 *
MUFA (g)	17.8	22.6	26.9	25.3	16.2	23.8	24.4	17.3	2.7	42.0	3.9	65.0	0.032 *	0.071	0.112
PUFA (g)	12.6	14.4	11.8	11.0	10.3	14.7	11.0	10.6	1.6	42.0	2.5	68.0	0.274	0.242	0.696
Cholesterol (mg)	22.1	124.1	295.5	273.3	3.8	20.0	254.9	24.5	44.0	42.0	47.2	83.0	<0.001 *	<0.001 *	0.001 *
Sugar (g)	59.0	77.4	83.2	94.3	58.3	69.0	77.8	85.2	10.2	42.0	15.4	64.0	0.254	0.124	0.928
Fibre (g)	20.9	20.3	16.2	17.9	18.2	25.0	14.6	21.1	2.2	42.0	3.0	71.0	0.486	0.044 *	0.059

SED^(a)^ and df^(a)^; standard error of difference and degrees of freedom for comparing Baseline and End means from the same dietary group. SED^(b)^ and df^(b)^; standard error of difference and degrees of freedom for comparing means from different dietary groups. In both cases, the SED values presented were for comparing a mean having the minimum number of reps to a mean with the maximum number and values are representative of the SED values for other comparisons of the same type. * Significance between dietary groups assessed by ANOVA (*p* ≤ 0.05). COH, carbohydrates; SFA, saturated fatty acids; MUFA, monounsaturated fatty acids; PUFA, polyunsaturated fatty acids.

**Table 3:** Average daily micronutrient intake non-inclusive of supplements of individuals in the 2019 cohort at baseline and end of study (End) provided Food Frequency Questionnaire (FFQ) estimates.

	Baseline (Mean)				End (Mean)				SED^(a)^	df^(a)^	SED^(b)^	df^(b)^	*p*-Value (Baseline-to-End)		
	Vegan (VV) (*n*12)	Vegetarian-Vegan (VegV) (*n*10)	Omnivore (OO) (*n*12)	Omnivore-Vegan (OV) (*n*12)	Vegan (VV) (*n*12)	Vegetarian-Vegan (VegV) (*n*10)	Omnivore (OO) (*n*12)	Omnivore-Vegan (OV) (*n*12)	Time × Diet		Time × Diet		Time	Diet	Time × Diet
Vitamin A (μg)	918.5	952.4	1147.0	1193.1	649.1	1122.3	906.3	839.8	201.0	42.0	285.5	67.0	0.061	0.670	0.287
Vitamin C (mg)	111.3	103.6	113.0	99.3	105.2	140.4	97.9	107.0	19.3	42.0	23.8	75.0	0.639	0.780	0.270
Thiamine (mg)	3.0	2.5	1.6	1.9	2.0	3.6	1.5	3.3	0.6	42.0	0.8	70.0	0.291	0.176	0.020 *
Riboflavin (mg)	1.1	1.5	1.7	1.6	1.0	1.5	1.4	1.3	0.2	42.0	0.3	60.0	0.021 *	0.221	0.730
Niacin (mg)	13.8	14.8	18.7	17.2	11.4	16.1	17.8	14.2	2.1	42.0	2.6	76.0	0.196	0.062	0.499
Vitamin B6 (mg)	1.6	1.7	2.0	2.0	1.4	1.8	1.8	1.7	0.2	42.0	0.3	70.0	0.203	0.270	0.588
Vitamin B12 (μg)	1.4	2.5	6.4	5.1	1.0	1.6	5.0	1.8	0.7	42.0	1.0	70.0	<0.001 *	<0.001 *	0.020 *
Folate (μg)	281.0	299.8	265.6	273.0	239.3	360.0	232.0	264.3	38.6	42.0	49.8	72.0	0.642	0.238	0.257
Calcium (mg)	651.3	825.1	852.6	879.7	535.9	802.7	657.2	609.8	99.9	42.0	151.0	64.0	0.003 *	0.387	0.344
Iron (mg)	10.6	11.3	9.9	10.7	9.0	12.0	9.2	10.1	1.1	42.0	1.6	66.0	0.287	0.448	0.580
Magnesium (mg)	307.8	323.4	276.7	314.3	267.2	359.1	253.4	309.9	31.3	42.0	47.8	63.0	0.513	0.320	0.368
Potassium (mg)	2680.3	3021.5	3043.6	3392.8	2386.1	3329.0	2709.2	2956.7	323.1	42.0	461.2	67.0	0.187	0.309	0.384
Sodium (mg)	1799.0	2173.3	2112.0	2193.2	1407.9	2146.9	1987.5	1853.5	193.3	42.0	337.0	58.0	0.019 *	0.277	0.490
Zinc (mg)	7.7	7.2	8.7	8.6	5.5	8.3	7.8	7.0	0.9	42.0	1.2	65.7	0.022 *	0.287	0.238
Selenium (μg)	35.6	41.3	56.0	52.0	25.3	34.0	49.4	29.9	4.7	42.0	6.8	66.0	<0.001 *	0.003 *	0.070
Iodine (μg)	28.3	61.4	125.7	125.2	18.7	39.2	105.1	38.2	14.3	42.0	18.0	74.0	<0.001 *	<0.001 *	<0.001 *
Vitamin D (μg)	1.8	2.4	3.5	2.7	1.6	1.5	2.7	1.7	0.4	42.0	0.7	56.0	<0.001 *	0.135	0.423

SED^(a)^ and df^(a)^; standard error of difference and degrees of freedom for comparing Baseline and End means from the same dietary group. SED^(b)^ and df^(b)^; standard error of difference and degrees of freedom for comparing means from different dietary groups. In both cases, the SED values presented were for comparing a mean having the minimum number of reps to a mean with the maximum number and values are representative of the SED values for other comparisons of the same type. * Significance between dietary groups assessed by ANOVA (*p* ≤ 0.05).

**Table 4:** Average daily iodine intake non-inclusive of supplements of the 2020 cohort at baseline and end of study (End) provided by iodine-specific Food Frequency Questionnaire (FFQ) estimates ^a^.

	Baseline (Mean)				End (Mean)				SED^(a)^	df^(a)^	SED^(b)^	df^(b)^	*p*-Value (Baseline-to-End)		
	Vegan (VV) (*n*14)	Vegetarian-Vegan (VegV) (*n*2)	Omnivore (OO) (*n*5)	Omnivore-Vegan (OV) (*n*13)	Vegan (VV) (*n*14)	Vegetarian-Vegan (VegV) (*n*2)	Omnivore (OO) (*n*5)	Omnivore-Vegan (OV) (*n*13)	Time × Diet		Time × Diet		Time	Diet	Time × Diet
Iodine (μg)	10.8	44.1	167.7	156.0	29.6	43.2	176.5	24.6	44.95	30.0	45.0	60.0	0.006 *	<0.001 *	<0.001 *
Foods Contributing to Iodine Intake from FFQ Output (μg day^−1^)															
Cow’s Milk (total)	-	5.4	38.7	30.6	-	0.0	51.9	0.4	14.9	12.2	19.6	33.0	0.040 *	0.017 *	0.071
Alternative Milk (total)	0.77	1.71	0.02	4.62	0.00	0.00	0.00	0.00	4.1	2.9	4.1	60.0	0.062	0.618	0.618
Dairy Yogurts	-	8.3	21.9	13.5	-	0.0	22.3	0.2	8.4	8.3	10.2	31.0	0.016 *	0.041 *	0.272
Non-Dairy Yogurts	2.6	5.3	0.1	2.8	2.2	11.2	1.1	0.8	3.2	2.4	3.3	60.0	0.685	0.050 *	0.362
Dairy Cheese	-	3.4	6.3	4.5	0.0	-	46.3	0.1	5.5	4.9	6.0	32.0	0.009 *	<0.001 *	<0.001 *
Dairy-Based Puddings	-	1.1	1.5	1.4	-	0.0	0.9	0.0	0.5	0.4	0.5	34.0	<0.001 *	0.145	0.336
Non-Dairy Puddings	1.8	2.9	0.1	5.0	2.2	4.7	0.1	1.4	5.0	0.6	5.0	60.0	0.482	0.645	0.675
Eggs (boiled, scrambled, etc.)	-	3.5	25.2	23.2	-	0.0	19.3	0.1	15.7	12.7	16.7	33.0	0.019*	0.311	0.442
Omelette	-	1.8	4.1	2.5	-	0.0	2.2	0.1	0.8	0.5	0.8	34.0	<0.001 *	<0.001 *	0.794
Egg-Based Deserts	-	1.1	2.6	4.0	-	0.0	10.5	0.2	4.5	3.2	4.5	34.0	0.119	0.746	0.054
White Fish	-	0.0	10.8	11.0	-	0.0	10.8	1.3	3.9	4.3	5.3	31.0	0.002 *	0.095	0.041 *
Oil-Rich Fish	-	0.0	17.0	14.8	-	0.0	8.3	0.6	13.5	11.5	15.0	33.0	0.060	0.608	0.735
Other Seafood	-	0.0	0.8	1.0	-	0.0	0.7	0.1	0.3	0.4	0.5	26.0	0.001 *	0.315	0.050 *
Seaweed	2.1	0.0	0.0	2.3	16.8	14.7	14.7	15.8	2.5	4.2	4.5	40.0	<0.001 *	0.875	0.921

^a^ Includes non-consumers of food groups. SED^(a)^ and df^(a)^; standard error of difference and degrees of freedom for comparing baseline and end means from the same dietary group. SED^(b)^ and df^(b)^; standard error of difference and degrees of freedom for comparing means from different dietary groups. In both cases, the SED values presented were for comparing a mean having the minimum number of reps to a mean with the maximum number and values are representative of the SED values for other comparisons of the same type. -, not applicable. * Significance between dietary groups assessed by ANOVA (*p* ≤ 0.05).

## Data Availability

The dietary data presented in this study are available on request from the corresponding author.

## Supplementary Materials

The following supporting information can be downloaded at: https://www.mdpi.com/article/10.3390/nu15234967/s1, Figure S1: Factors influencing food choice; results from the Food Choice Questionnaire (FCQ) in 2019 and 2020; Table S1: Questions assessing micronutrient knowledge in the 2019 cohort; Table S2 Questions assessing iodine knowledge in the 2020 cohort; Table S3: Motivation towards following a vegan diet for individuals recruited in 2019; Table S4: Motivation towards following a vegan diet for individuals recruited in 2020; Table S5: Average micronutrient intake provided by dietary supplements recorded in Food Frequency Questionnaire (FFQ) in the 2019 cohort at baseline and end, compared with 10% of the Reference Nutrient Intake (RNI); Table S6: Average micronutrient intake provided by dietary supplements recorded in the Food Frequency Questionnaire (FFQ) in the 2020 cohort at baseline and end, compared with 10% of the Reference Nutrient Intake (RNI); Table S7: Baseline micronutrient knowledge in the 2019 cohort; Table S8: Baseline record of iodine knowledge (%) by dietary group in 2020.
