# Supplementary material for: Adoption of a Short-Term (4-Week) Vegan Diet as Part of ‘Veganuary’ Significantly Reduces Saturated Fatty Acid (SFA), Cholesterol, B12, and Iodine Intake in Omnivorous Individuals—An Observational Study
Source: Nutrients. 2023 Nov 30;15(23):4967. doi: 10.3390/nu15234967 (PMC10708059; doi:10.3390/nu15234967)
Supplement: Supplementary file 1 [file nutrients-15-04967-s001.zip › nutrients-2689146-supplementary.pdf]

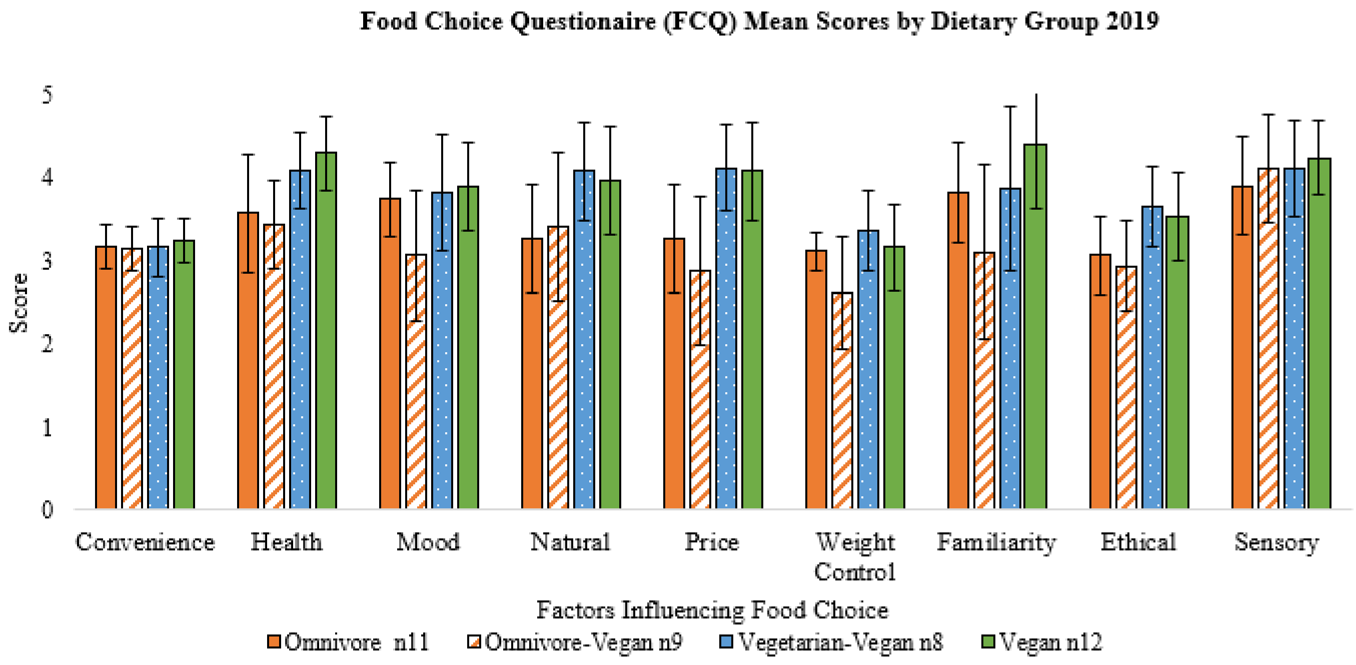

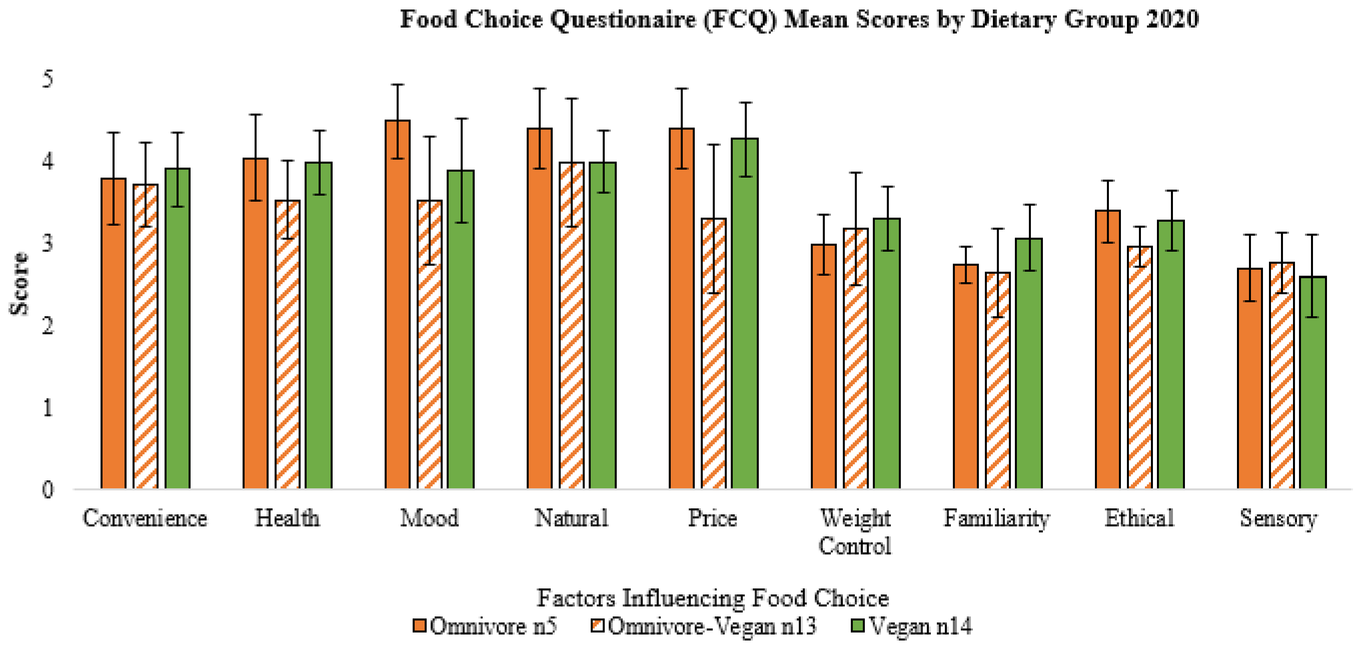

Figure S1. Factors influencing food choice; results from the Food Choice Questionnaire (FCQ) in 2019 and 2020.

Table S1. Questions assessing micronutrient knowledge in the 2019 cohort.

Question
1. Before you volunteered to take part in this study were you aware of any symptoms associated with micronutrient deficiencies?
1. A. If Yes, what deficiency symptoms are you aware of? Please list all of the symptoms you are aware of. Be as specific as possible.
2. Are you confident that your current diet provides adequate micronutrients?

Table S2. Questions assessing iodine knowledge in the 2020 cohort.

Question	Responses
1. Are you aware of the nutrient iodine?	Yes<br>No
2. Which of the following do you think is the richest food source of iodine in the UK?	Fruit<br>Vegetables<br>Eggs<br>Meat and meat products<br>Salt (table)<br>Milk and Dairy<br>Bread<br>Dairy-alternative or milk substitute<br>Fish and seafood<br>Nuts and seeds<br>Breakfast cereals<br>Non-dairy yogurt and cheese<br>Do not know
3. What do you understand to be the main health problem/s associated with not having enough iodine in the diet? (select all that apply)	Weak bones and teeth<br>Heart conditions<br>Malformations in pregnancy (Birth defects)<br>Impaired physical development during childhood<br>Neural Tube Defects<br>Allergy<br>Do not know<br>Blindness<br>Goitre<br>Weak immune system<br>Mental Retardation<br>Arthritis<br>Other (free text available)
4. Based on your understanding, at which stage of the life cycle do you think iodine is most important?	Babies and young children<br>Teenagers<br>Adults<br>Elderly adults<br>Pregnant and breastfeeding women<br>Women of childbearing age<br>Don't know
5. Do you think iodine deficiency is a common health problem in the UK and Ireland now?	Yes<br>No
6. Do you think that your present diet provides you with enough iodine?	Yes<br>No<br>Unsure
7. Do you plan your diet to ensure you eat foods rich in iodine?	Yes<br>No<br>Unsure
8. Have you ever received any information related to iodine and health?	Yes (free text available)<br>No<br>Unsure

Table S3. Motivation towards following a vegan diet for individuals recruited in 2019 Higher mean (SD) scores relate to greater motivation for each trait (max score 5).

Motivation for a vegan diet Mean (SD)	Vegan (VV) (*n*24)	Vegetarian-vegan (VegV) (*n*14)	Omnivore (OO) (*n*21)	Omnivore-vegan (OV) (*n*22)	P
Health	3.8 (1.2)	4.1 (0.9)	3.5 (1.3)	3.7 (1.2)	0.539
Factory farming	4.2 (1.0)	4.3 (1.0)	2.6 (1.0)	2.9 (1.4)	<.001*
Sustainability	4.6 (0.7)	4.4 (0.6)	3.0 (1.4)	3.6 (1.2)	<.001*
Climate protection	4.4 (0.7)	4.4 (0.6)	3.2 (1.4)	3.6 (1.2)	<.001*
Vegan friends	2.0 (1.2)	2.7 (1.4)	1.4 (0.6)	1.7 (0.9)	0.005*
Food scandals	2.1 (1.2)	2.5 (1.1)	1.8 (0.6)	1.6 (1.0)	0.048*
Animal welfare	4.2 (1.0)	4.3 (1.1)	2.9 (1.0)	3.0 (1.2)	<.001*
Food safety	2.8 (1.2)	3.5 (1.3)	2.7 (1.2)	2.2 (1.2)	0.017*
Food availability	2.3 (1.3)	3.1 (1.3)	2.5 (1.1)	2.2 (1.2)	0.130
Food choice	2.3 (1.2)	3.3 (1.2)	2.4 (1.1)	2.1 (1.2)	0.016*
Food trends	1.7 (1.0)	2.2 (1.6)	1.7 (0.8)	1.9 (1.3)	0.462
Food taste	2.5 (1.4)	3.8 (1.3)	3.4 (1.3)	2.4 (1.4)	0.004*
Vegan family	1.6 (1.1)	2.0 (1.5)	1.3 (0.6)	1.4 (0.9)	0.177
Personal morals	4.1 (1.1)	4.3 (1.0)	2.3 (1.0)	2.7 (1.4)	<.001*
Rotten meat	2.0 (1.4)	2.5 (1.4)	2.3 (1.5)	1.7 (1.3)	0.360
Religion	1.3 (0.7)	1.3 (1.0)	1.2 (0.7)	1.0 (0.2)	0.618
New year resolution	1.6 (1.1)	1.9 (1.5)	1.3 (0.5)	2.0 (1.1)	0.193
Previous experience	1.5 (0.9)	3.0 (1.7)	2.5 (1.0)	1.8 (1.3)	0.001*
Vegan Society support	2.2 (1.3)	2.9 (1.6)	1.2 (0.4)	1.6 (1.0)	<.001*
Challenge	2.2 (1.4)	3.3 (1.5)	1.8 (0.9)	3.2 (1.3)	<.001*
Cost or expense	1.8 (1.0)	2.0 (1.4)	2.6 (1.3)	2.0 (1.3)	0.156
Food variety	2.1 (1.2)	2.6 (1.5)	2.5 (1.3)	2.2 (1.4)	0.648
Novelty	1.3 (0.7)	1.9 (1.4)	2.0 (1.3)	2.3 (1.3)	0.029*
Social media	1.9 (1.3)	2.1 (1.3)	1.4 (0.7)	1.6 (1.2)	0.197
Weight loss	1.4 (0.9)	2.5 (1.2)	1.9 (1.2)	2.5 (1.5)	0.011*
Media and TV	2.8 (1.4)	2.7 (1.5)	1.5 (0.7)	2.0 (1.5)	0.197

*Significance between dietary groups assessed by ANOVA (P = < 0.05).

Table S4. Motivation towards following a vegan diet for individuals recruited in 2020 Higher mean (SD) scores relate to greater motivation for each trait (max score 5).

Motivation for a vegan diet Mean (SD)	Vegan (VV) (*n*45)	Vegetarian-vegan (VegV) (*n*6)	Omnivore (OO) (*n*6)	Omnivore-vegan (OV) (*n*16)	P
Health	3.1 (1.4)	3.2 (1.5)	3.2 (1.3)	2.5 (1.3)	0.584
Factory farming	4.4 (1.0)	4.0 (0.9)	3.5 (1.7)	2.6 (1.4)	0.002*
Sustainability	4.2 (1.0)	4.5 (1.3)	3.7 (1.5)	3.8 (1.4)	0.500
Climate protection	4.3 (1.1)	3.8 (1.2)	3.5 (1.4)	3.8 (1.5)	0.301
Vegan friends	1.8 (1.1)	2.3 (1.4)	1.3 (0.5)	1.8 (1.0)	0.523
Food scandals	2.5 (1.3)	2.7 (1.0)	2.8 (1.8)	1.5 (0.9)	0.063
Animal welfare	4.8 (0.7)	4.0 (1.1)	2.8 (1.2)	3.3 (1.5)	0.001*
Food safety	2.1 (1.4)	2.5 (1.4)	2.5 (1.5)	2.0 (1.4)	0.887
Food availability	2.0 (1.2)	2.7 (1.5)	2.3 (1.2)	1.5 (1.2)	0.596
Food choice	2.2 (1.3)	2.3 (1.4)	3.5 (1.3)	2.0 (1.4)	0.551
Food trends	1.4 (0.8)	2.3 (1.0)	1.8 (1.0)	1.6 (0.7)	0.055
Food taste	2.2 (1.3)	2.8 (1.5)	2.8 (1.8)	2.4 (1.6)	0.125
Vegan family	1.9 (1.3)	2.7 (1.9)	1.3 (0.5)	1.6 (1.0)	0.149
Personal morals	4.6 (0.9)	3.8 (0.4)	2.5 (1.4)	3.5 (1.5)	0.001*
Rotten meat	1.7 (1.0)	2.2 (1.6)	1.7 (1.2)	1.5 (1.0)	0.711
Religion	1.0 (0.0)	1.2 (0.4)	2.0 (0.0)	1.0 (0.0)	0.160
New year resolution	1.3 (0.8)	2.0 (1.7)	2.5 (0.8)	1.6 (1.2)	0.210
Previous experience	1.3 (0.8)	2.5 (1.8)	1.5 (0.8)	1.5 (0.9)	0.058
Vegan Society support	2.1 (1.5)	2.3 (1.5)	1.2 (0.4)	1.4 (0.9)	0.167
Challenge	1.6 (0.9)	2.0 (1.1)	2.5 (1.3)	2.9 (1.5)	0.005*
Cost or expense	1.4 (0.8)	2.5 (1.4)	3.2 (1.3)	1.2 (0.5)	0.001*
Social media	1.7 (1.0)	2.2 (1.0)	1.3 (0.5)	1.4 (1.0)	0.297
COVID-19 infection	1.2 (0.7)	1.8 (1.3)	1.8 (1.2)	1.1 (0.5)	0.001*
Disease outbreaks (in general)	1.4 (1.1)	2.0 (1.6)	2.2 (1.5)	1.1 (0.3)	0.007*

*Significance between dietary groups assessed by ANOVA (P = < 0.05).

Table S5. Average micronutrient intake provided by dietary supplements recorded in Food Frequency Questionnaire (FFQ) in the 2019 cohort at baseline and end, compared with 10% of the Reference Nutrient Intake (RNI).

	Vegan (VV) (*n*12)			Vegetarian-vegan (VegV) (*n*10)			Omnivore (OO) (*n*12)			Omnivore-vegan (OV) (*n*12)			50% of RNI	
	n	Mean	SD	*n*	Mean	SD	*n*	Mean	SD	*n*	Mean	SD	Female	Male
**Baseline**														
Vitamin A (μg)	4	1000.0	400.0	2	800.0	0.0	3	633.3	288.7	2	600.0	282.8	300.0	350.0
Vitamin C (mg)	4	85.0	5.8	2	85.0	7.1	3	199.0	140.1	2	198.6	224.2	20.0	20.0
Thiamine (mg)	5	1.7	0.8	3	1.8	0.7	2	7.3	6.7	1	8.4	0.0	0.4	0.5
Riboflavin (mg)	5	2.0	0.8	3	2.1	0.7	2	3.8	1.4	1	10.7	0.0	0.6	0.7
Niacin (mg)	4	16.8	1.1	3	13.7	5.8	2	18.0	0.0	2	7.7	0.4	6.5	8.5
Vitamin B6 (mg)	6	2.9	2.4	3	2.9	2.7	2	8.0	2.8	3	18.9	27.1	0.6	0.7
Vitamin B12 (μg)	9	6.9	4.3	4	6.6	5.5	2	5.8	4.6	4	6.2	3.2	0.8	0.8
Folate (μg)	7	214.3	69.0	3	185.7	122.1	2	350.0	70.7	-	-	-	100.0	100.0
Calcium (mg)	4	300.0	200.0	3	138.1	107.2	1	200.0	0.0	-	-	-	400.0	350.0
Iron (mg)	5	25.0	24.6	3	14.0	0.0	2	20.0	5.7	-	-	-	7.4	4.4
Magnesium (mg)	1	100.0	0.0	3	71.4	49.5	2	214.3	69.0	-	-	-	135.0	150.0
Zinc (mg)	3	13.3	2.9	3	8.8	6.9	2	15.0	0.0	1	5.0	0.0	3.5	4.8
Selenium (μg)	2	42.5	17.7	1	55.0	0.0	1	150.0	0.0	-	-	-	30.0	37.5
Iodine (μg)	3	125.0	43.3	1	150.0	0.0	1	100.0	0.0	1	75.0	0.0	70.0	70.0
Vitamin D (μg)	8	8.4	3.5	4	8.8	2.5	6	7.9	4.6	4	25.0	33.4	5.0	5.0
**End**														
Vitamin A (μg)	2	800.0	0.0	6	685.7	279.9	2	800.0	0.0	3	833.3	57.7	300.0	350.0
Vitamin C (mg)	2	85.0	7.1	3	105.9	90.1	1	90.0	0.0	3	90.0	0.0	20.0	20.0
Thiamine (mg)	2	1.8	1.0	8	3.7	5.8	1	2.5	0.0	4	4.1	4.0	0.4	0.5
Riboflavin (mg)	2	2.1	1.0	8	2.9	3.0	1	2.8	0.0	4	5.5	6.4	0.6	0.7
Niacin (mg)	2	17.0	1.4	8	18.2	14.1	1	18.0	0.0	4	19.3	3.9	6.5	8.5
Vitamin B6 (mg)	3	2.8	2.8	8	3.4	2.6	1	6.0	0.0	5	24.7	42.2	0.6	0.7
Vitamin B12 (μg)	8	173.8	70.0	9	3.8	3.6	1	2.5	0.0	5	17.6	19.3	0.8	0.8
Folate (μg)	3	200.0	100.0	8	223.2	128.4	1	300.0	0.0	4	416.8	173.4	100.0	100.0
Calcium (mg)	2	200.0	0.0	7	149.0	87.2	1	200.0	0.0	4	232.5	116.4	400.0	350.0

Iron (mg)	3	14.0	0.0	6	12.0	4.9	1	14.0	0.0	3	15.33	2.3	7.4	4.4
Magnesium (mg)	1	100.0	0.0	5	65.7	46.9	-	-	-	1	42.0	0.0	135.0	150.0
Zinc (mg)	2	12.5	3.5	7	9.7	6.1	1	15.0	0.0	3	13.7	2.3	3.5	4.8
Selenium (µg)	2	42.5	17.7	3	39.3	27.2	-	-	-	1	55.0	0.0	30.0	37.5
Iodine (µg)	3	125.0	43.3	4	117.9	64.3	-	-	-	1	150.0	0.0	70.0	70.0
Vitamin D (µg)	6	8.8	2.1	7	6.6	3.4	4	12.5	2.9	6	19.6	27.9	5.0	5.0

-; Not applicable

Table S6. Average micronutrient intake provided by dietary supplements recorded in the Food Frequency Questionnaire (FFQ) in the 2020 cohort at baseline and end, compared with 10% of the Reference Nutrient Intake (RNI).

	Baseline										
	Vegan (VV) (*n*14)			Vegetarian-vegan (VegV) (*n*3)			Omnivore-vegan (OV) (*n*13)			50% of RNI	
	n	Mean	SD	*n*	Mean	SD	*n*	Mean	SD	Female	Male
Vitamin A (μg)	3	633.3	288.7	1	800.0	0.0	2	600.0	282.8	300.0	350.0
Vitamin C (mg)	4	65.0	19.2	1	80.0	0.0	2	46.0	48.1	20.0	20.0
Thiamine (mg)	3	4.1	5.1	1	1.1	0.0	2	6.1	6.3	0.4	0.5
Riboflavin (mg)	3	2.6	2.1	1	5.0	0.0	2	7.8	8.1	0.6	0.7
Niacin (mg)	3	22.7	11.6	1	16.0	0.0	2	30.5	20.5	6.5	8.5
Vitamin B6 (mg)	4	3.3	4.5	1	2.0	0.0	2	4.8	3.8	0.6	0.7
Vitamin B12 (μg)	12	120.8	295.3	1	30.0	0.0	4	19.7	21.0	0.8	0.8
Folate (μg)	3	240.0	144.2	1	200.0	0.0	2	380.0	28.3	100.0	100.0
Calcium (mg)	1	200.0	0.0	1	200.0	0.0	2	260.0	198.0	400.0	350.0
Iron (mg)	3	14.0	0.0	1	14.0	0.0	1	14.0	0.0	7.4	4.4
Magnesium (mg)	2	63.5	51.6	1	60.0	0.0	3	102.6	53.0	135.0	150.0
Zinc (mg)	2	12.5	3.5	1	10.0	0.0	2	9.5	0.7	3.5	4.8
Selenium (μg)	2	77.5	31.8	1	100.0	0.0	1	55.0	0.0	30.0	37.5
Iodine (μg)	2	150.0	0.0	1	150.0	0.0	1	150.0	0.0	70.0	70.0
Vitamin D (μg)	8	20.9	22.1	1	20.0	0.0	7	12.5	7.0	5.0	5.0
	End										
Vitamin A (μg)	3	800.0	0.0	2	800.0	0.0	5	800.0	0.0	300.0	350.0
Vitamin C (mg)	4	75.0	10.0	2	85.0	7.1	5	82.0	2.5	20.0	20.0
Thiamine (mg)	4	3.3	4.5	2	1.8	1.0	5	1.5	0.6	0.4	0.5
Riboflavin (mg)	4	2.3	1.8	2	2.1	1.0	5	1.8	0.6	0.6	0.7
Niacin (mg)	4	21.0	0.0	2	17.0	1.4	5	16.4	0.9	6.5	8.5
Vitamin B6 (mg)	4	3.6	4.3	2	3.7	3.3	4	2.7	2.2	0.6	0.7
Vitamin B12 (μg)	11	85.9	172.6	2	10.0	10.6	8	11.3	19.9	0.8	0.8
Folate (μg)	4	250.0	100.0	2	250.0	70.7	5	260.0	89.4	100.0	100.0
Calcium (mg)	1	200.0	0.0	2	200.0	0.0	5	184.0	35.8	400.0	350.0
Iron (mg)	3	14.0	0.0	2	14.0	0.0	5	14.0	0.0	7.4	4.4

Magnesium (mg)	2	63.5	51.6	1	27.0	0.0	3	37.0	17.3	135.0	150.0
Zinc (mg)	2	20.0	7.1	2	12.5	3.5	4	11.3	2.5	3.5	4.8
Selenium (µg)	2	77.5	31.8	2	55.0	0.0	3	55.0	0.0	30.0	37.5
Iodine (µg)	2	150.0	0.0	1	150.0	0.0	3	150.0	0.0	70.0	70.0
Vitamin D (µg)	8	17.5	15.6	2	7.5	3.5	9	19.1	30.8	5.0	5.0

Table S7. Baseline micronutrient knowledge in the 2019 cohort.

	Dietary group				
	Vegan (VV) (*n*24)	Vegetarian-vegan (VegV) (*n*15)	Omnivore (OO) (*n*20)	Omnivore-vegan (OV) (*n*23)	*P*
Self-assessed micronutrient Awareness					
Yes (%)	4.2	20.0	30.0	26.1	0.098
No (%)	95.8	80.0	70.0	73.9	
Awareness of Specific Micronutrients					
Vitamin A (%)	25.0	13.3	35.0	34.8	0.447
Vitamin C (%)	45.8	26.7	50.0	43.5	0.573
Niacin (%)	4.2	0.0	0.0	0.0	-
Vitamin B6 (%)	4.2	0.0	0.0	0.0	-
Vitamin B12 (%)	50.0	33.3	20.0	8.7	0.012*
Folate (%)	4.2	0.0	5.0	0.0	-
Calcium (%)	33.3	26.7	40.0	30.4	0.865
Iron (%)	54.2	53.3	35.0	43.5	0.587
Magnesium (%)	8.3	0.0	15.0	8.7	0.543
Potassium (%)	12.5	6.7	15.0	13	0.967
Sodium (%)	0.0	6.7	5.0	8.7	0.549
Zinc (%)	12.5	13.3	10.0	17.4	0.943
Selenium (%)	8.3	6.7	5.0	4.3	1.000
Iodine (%)	20.8	6.7	15.0	17.4	0.753
Vitamin D	45.8	33.3	50.0	47.8	0.796
Awareness of Micronutrient Deficiencies					
Yes (%)	58.3	66.7	55.0	65.2	0.216
No (%)	41.7	33.3	45.0	34.8	
Non-Specific Deficiency Symptom (%)	16.7	12.3	4.2	4.3	0.882
Vitamin A (%)	10.0	13.3	4.2	4.3	0.650
Vitamin C (%)	20.8	13.3	25.0	21.7	0.873
B Vitamins (non-inclusive of B12)					1.000
(%)	8.3	6.7	5.0	8.7	
Vitamin B12 (%)	25	6.7	20.0	0.0	0.032*
Folate (%)	0.0	0.0	5.0	4.3	-
Calcium (%)	0.0	0.0	5.0	17.4	-
Iron (%)	33.3	33.3	20.0	8.7	0.148
Magnesium (%)	0.0	6.7	0.0	0.0	-
Zinc (%)	8.3	0.0	5.0	0.0	-
Iodine (%)	4.2	6.7	20.0	13	0.377
Vitamin D (%)	12.5	26.7	20.0	21.7	0.709

Fisher-Freeman-Halton Exact Test (P < 0.05).
-; Not applicable analysis for more than two groups had counts less than 1.

Table S8. Baseline record of iodine knowledge (%) by dietary group in 2020.

Responses	Vegan (VV) (*n*45)	Vegetarian-vegan (VegV) (*n*6)	Omnivore (OO) (*n*6)	Omnivore-vegan (OV) (*n*16)	*P*
Awareness of the importance of adequate dietary intake					
Yes	75.6	33.3	83.3	87.5	0.692
No	24.4	66.7	16.7	12.5	
Symptoms correctly identified					
Yes	33.3	33.3	66.7	62.5	<.001*
No	66.7	66.7	33.3	37.5	
Knowledge of iodine deficiency symptoms					
Mental Retardation	8.9	16.7	33.3	18.8	0.206
Malformations in pregnancy (Birth defects)	13.3	16.7	33.3	18.8	0.649
Goitre /enlargement of the thyroid gland	11.1	33.3	33.3	50.0	0.008*
Impaired physical development during childhood	15.6	16.7	16.7	25.0	0.751
Neural Tube Defects	6.7	0.0	16.7	0.0	0.371
Heart conditions	11.1	0.0	16.7	18.6	0.584
Weak immune system	20.0	66.7	16.7	37.5	0.062
Blindness	0.0	0.0	16.7	0.0	-
Weak bones and teeth	6.7	33.3	0.9	12.5	0.180
Arthritis	2.2	0.0	0.0	0.0	-
Other	8.9	0.0	0.0	0.0	-
Do not know/ no response	48.9	33.3	33.3	12.5	0.089
Understanding of iodine in the life cycle					
Elderly adults	2.2	0.0	0.0	0.0	-
Adults	0.0	0.0	0.0	25.0	-

Pregnant and breastfeeding women	9.0	0.0	2.0	7.0	0.116
Women of childbearing age	0.0	0.0	33.3	0.0	-
Teenagers	15.6	0.0	0.0	0.0	-
Babies and young children	15.6	33.3	16.7	12.5	0.614
Don't know	46.7	66.7	16.7	18.8	0.065
Perceptions of the main food sources of iodine in the UK diet					
Cow's milk and dairy	2.2	0.0	33.3	18.8	0.021*
Fish and seafood	35.6	66.7	66.7	43.8	0.292
Eggs	2.2	0.0	0.0	6.2	0.623
Meat and meat products	4.4	0.0	0.0	0.0	-
Nuts and seeds	0.0	0.0	0.0	12.5	-
Table salt	0.0	0.0	0.0	6.2	-
Vegetables	17.8	0.0	0.0	6.2	0.499
Don't know	26.7	33.3	0.0	6.2	0.133
Knowledge of iodine deficiency being a problem in the UK					
Yes	33.3	0.0	50.0	81.2	<.001*
No	15.6	33.3	16.7	6.2	
Don't know	51.1	66.7	33.3	12.5	
Reported planning their diet to include sources of iodine					
Yes	6.7	0.0	100.0	81.2	<.001*
No	75.6	66.7	0.0	6.2	
Unsure or don't know	17.8	33.3	0.0	12.5	
Currently receiving iodine information					
Yes	6.7	0.0	16.7	18.8	<.001*
No	88.9	100.0	83.3	81.2	
Unsure or don't know	4.4	0.0	0.0	0.0	
Total iodine knowledge score (mean)					
Total score	1.4	1.5	3.0	2.8	0.004*

Fisher-Freeman-Halton Exact Test ($P < 0.05$).
-; Not applicable analysis for more than two groups had counts less than 1.
